# ACE/ACE2 Ratio: A Key Also in 2019 Coronavirus Disease (Covid-19)?

**DOI:** 10.3389/fmed.2020.00335

**Published:** 2020-06-18

**Authors:** Pasquale Pagliaro, Claudia Penna

**Affiliations:** Department of Clinical and Biological Sciences, University of Torino, Turin, Italy

**Keywords:** RAS, comorbidities, macrophage, exercise, ARDS (acute respiratory distress syndrome), hypoxia

## Introduction

Angiotensin-converting enzyme 2 (ACE2) is an aminopeptidase that converts Angiotensin (Ang) II into Ang (1-7). Coronavirus uses ACE2 as a cellular receptor to invade target cells. In particular, the spike protein of SARS-CoV-2 (the beta-coronavirus responsible for Covid-19) is processed by transmembrane protease-serine 2 (TMPRSS2) and favors the binding of the spike protein to ACE2 (1–3).

**Figure 1:**
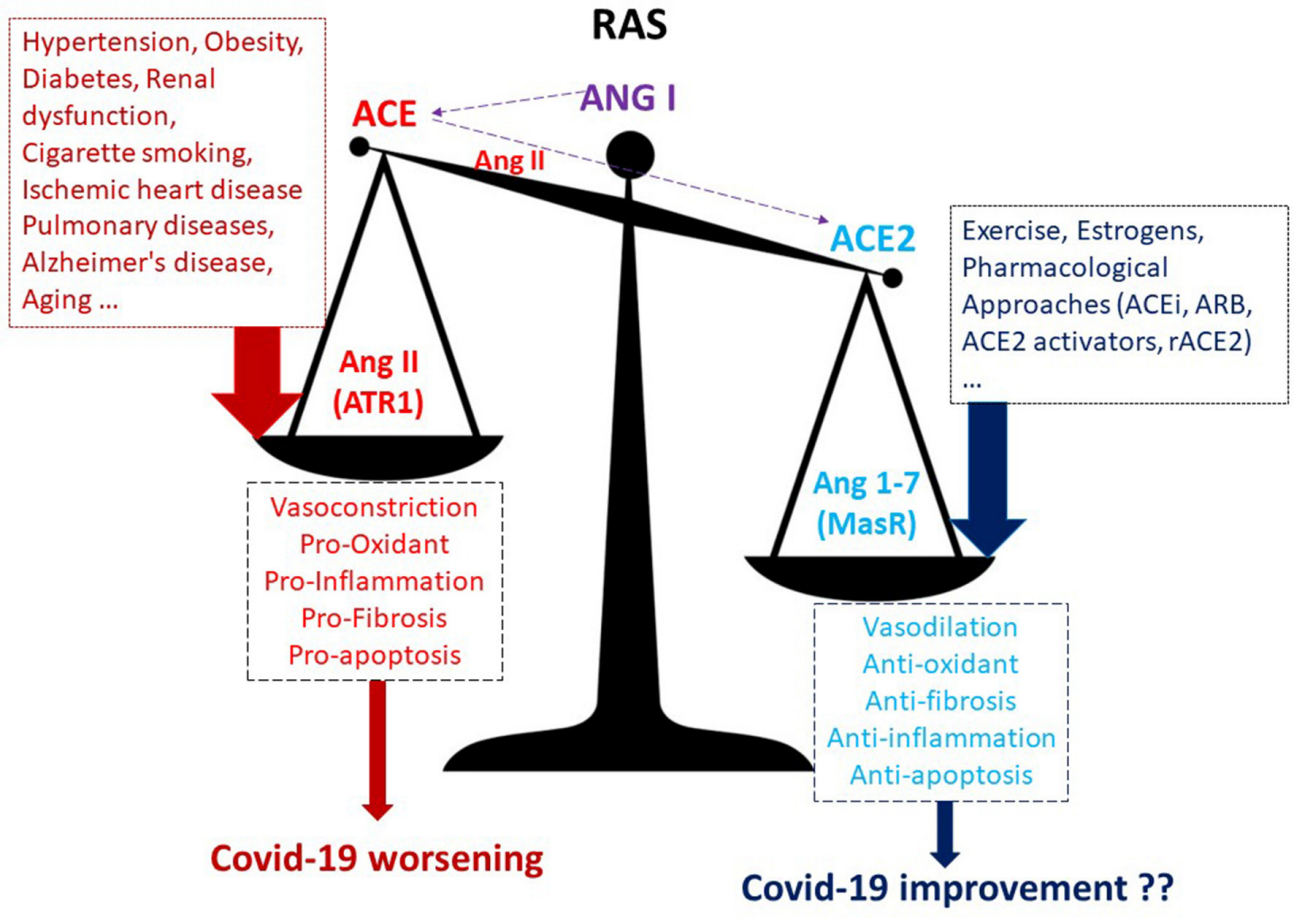
A higher ACE/ACE2 ratio may increase the risk of worse outcomes in Covid-19 infection. ACEi, ACE inhibitors; ARB, AT1R blockers; ACE, angiotensin-converting enzyme; MasR, Mas receptor; rACE2, recombinant ACE2; ATR1, angiotensin receptor 1.

It is well-known that Ang II, acting on AT1 receptors, exerts powerful vasoconstrictor, pro-fibrotic, and pro-inflammatory effects. In contrast, Ang (1-7), acting on Mas receptors (MasR), is a potent vasodilator, anti-apoptotic, and anti-proliferative agent (Figure 1). Therefore, ACE2 is a negative regulator of classical ACE in the renin-angiotensin system (RAS). The two enzymes are involved in maintaining the homeostasis of RAS and in regulating blood pressure as well as the fluid and salt balance. The human ACE2 gene is located on chromosome Xp22. Moreover, the ACE/ACE2 activity ratio in females is lower than that in the male serum. This different ratio may be partially attributed to the two X chromosomes and to estrogens effect on ACE2 activity (4). In both sexes, ACE2 is largely expressed in lungs, liver, intestine, brain, heart, and kidneys, and also in testes. In almost all the pathological conditions, especially those of the cardiovascular system, there is an increase in the ACE/ACE2 ratio within the organs and systems (5–9). This ACE/ACE2 imbalance is very often due to a downregulation of ACE2 levels, and this ratio alteration is accompanied by disturbance in RAS homeostasis. For instance, it has been found that the ACE/ACE2 ratio is high in the glomeruli in the high-salt diet animals, and it is accompanied by renal dysfunction and oxidative stress (5). Also, in the heart, a high-glucose diet upregulated ACE and downregulated ACE2, leading to the augmentation of ACE/ACE2 ratio (6). Moreover, downregulation of ACE2 has been described in pulmonary arterial hypertension and cigarette smoker patients (7). The ACE/ACE2 ratio increase was also correlated with the systolic blood pressure, the serum creatine level, the fasting blood glucose level, and the proteinuria in humans (8). ACE2 is reduced, and the ACE/ACE2 ratio increased also in Alzheimer's disease in association with increasing amyloid-β and tau pathology. (9). Notably, SARS-CoV-2, which binds with ACE2 to enter the targeted cells, also leads to downregulation of ACE2. All in all, it seems that when ACE2 levels or activity are low and the ACE/ACE2 ratio increases we are in trouble (Figure 1) and may be more at risk of having a worse outcome in Covid-19 infection.

## COVID-19 and Comorbidities

The Italian ISS (https://www.epicentro.iss.it/coronavirus/sars-cov-2-decessi-italia, accessed on April 26th 2020) reports that among 23,188 SARS-CoV-2 patients dying in Italy, 3.6% patients presented with no comorbidities, 14.4% with a single comorbidity, 21.1% with two, and 60.9% with three or more comorbidities. Among these comorbidities, the most represented is hypertension (69.1%), followed by ischemic heart disease (27.5%), chronic renal failure (21.1%), atrial fibrillation (22%), pulmonary diseases (17.1%), heart failure (16.1%), and some other comorbidities with <15% incidence. Of note is that all these pathologies are characterized by themselves by a downregulation of ACE2 and a high ACE/ACE2 ratio (10–14). The majority of deceased patients were aged (over 60) and obese (in the Italian report, obesity is present in 12.2% of deceased patients). In addition, these two conditions are characterized by an increasing ACE/ACE2 ratio (15, 16). Therefore, we wonder whether the invasion by SARS-CoV-2 and the downregulation of ACE2 are jointly responsible for a high incidence of dramatic acute respiratory distress syndrome (ARDS), cardiovascular complications, and high lethality of Covid-19. Is it worthwhile to try to re-establish an appropriate ACE/ACE2 ratio?

## If You Cannot Breathe, Nothing Else Matters

It has been reported that SARS-CoV-2 has an affinity for ACE2 that is 10 times higher in comparison to SARS-CoV's affinity for this enzyme (2). Is ACE2 like a Trojan horse (1)? Is it a gift of nature that also allows the enemy to enter into cells? Should we therefore say “*timeo Danaos et dona ferentes*”? We believe ACE2 is not an enemy. We believe it is almost an innocent witness to the crime, and we will present here some clues to exculpate it. In our opinion, ACE2 is the key for the virus to enter the organism, but it is not responsible for the injury determined by the virus.

Of course, organs that express a high level of ACE2 are the targets of SARS-CoV-2 infection. This virus diffuses and is transmitted through human respiratory droplets. Therefore, the lung is the principal and initial target organ of SARS-CoV-2 infection (3). The subsequent pathogenic mechanisms are not strictly correlated with neither the number/expression of ACE2 and its activity nor with the viremia. For instance, a correlation between viremia and ARDS in patients with severe Covid-19 has not been observed (17). Moreover, estrogen shifts the system toward the ACE2/Ang 1-7 formation and ACE2 activity is higher in female than that in the male serum (18); however, the worst and most lethal Covid-19 infections occur predominantly in males [the Italian ISS (https://www.epicentro.iss.it/coronavirus/sars-cov-2-decessi-italia, accessed on April 26th 2020) reports that among 23,188 SARS-CoV-2 patients dying in Italy, women are 8,500 (36.7%)]. We believe that the subsequent inflammation and cytokine storm is responsible for the *primum movens* for Covid-19 worsening rather than viremia. For instance, viroporins-induced NLRP3/inflammasome activation and excessive production of IL-1β may be important pathological mechanisms (19). After the virus enters the cells, ACE2 is likely to decrease its activity, thus favoring an increase of the ACE/ACE2 balance toward the prevalence of ACE arm in the RAS. First of all, the prevalence of ACE arm determines a direct increase of ROS production, vasoconstriction, and inflammation. Indeed, Ang II action on its AT1 receptors promotes NAD(P)H oxidase upregulation, oxidative stress, and cytokine production (20). Cytokine storm, ROS, and inflammation lead to vascular permeability, diffuse alveolar damage, pulmonary edema, and, eventually, to ARDS (21, 22). Which cells in the lungs mediate this inflammatory response? It is likely that, among other cells, macrophages may play a pivotal role. Indeed, macrophages express ACE2 receptors (23), and three different macrophages populations have been described by Tan and Krasnow (24) within the respiratory tract. These three populations may respond differently to virus infection, and the different representation of these macrophages may explain the range of clinical scenarios from asymptomatic, to paucisymptomatic, and to dramatic pneumonia. This is a hypothesis that need to be ascertained. Nevertheless, when the cytokine storm starts and edema/ARDS ensue, hypoxia occurs, which may exacerbate vasoconstriction, through the typical hypoxia-induced vasoconstriction in the pulmonary vessels [for more details on cytokine storm the reader is redirected to (19, 25)]. For some organs, such as the brain and heart, hypoxia represents an intolerable condition that may lead to lethal outcomes. Together a marked increase in macrophage infiltration, hypoxia can mediate the myocardial damage that accompanies the Covid-19 infection. In the heart, multiple different macrophage subtypes have recently been identified (26), and they can mediate the infection-induced injury. From autopsies, it appears that only a third of patients who died for cardiovascular complications, among Covid-19 patients, have evidence of coronaviruses inside the myocardium (27). This is another sign that it is not important how much virus enters but how the organism reacts to the virus.

Chronic hypoxia-driven vasoconstriction contributes significantly to pulmonary hypertension and several pulmonary hypertension-related diseases, including edema, right heart failure, and myocardial ischemic events (28). Paradoxically, hypoxia may exacerbate redox stress through at least two mechanisms: hypoxia-induced hyperventilation and subsequent alkalosis and dysregulation of iron metabolism (29–32). Pulmonary edema hypoxia is not easy to treat. Indeed, oxygen therapy remains the major life-saving concern in intensive care unit (ICU). In ICU-patients, excess oxygen delivery may cause considerable harm in which redox stress plays a pivotal role (33–35). Therefore, additional therapies that limit redox stress and inflammation are needed, including those aimed at improving the ACE/ACE2 ratio.

## Approaches to Improve Ace/Ace2 Ratio

All the above data support the idea that an imbalance in the ACE/ACE2 ratio may be a predisposing cause to the worsening of the Covid-19. It has also been suggested that the increased concentration of ACE2 receptors in in the lungs of children may have a protective effect on severe clinical manifestations due to SARS-CoV-2 invasion (36). Also, these data support a negative correlation between ACE2 expression and Covid-19 severe outcomes. Perhaps, therapies improving this ratio may be useful in infected patients (37–40). The RAS is quite complex, and several pharmacological approaches are under evaluation to benefit from ACE downregulation and ACE2 upregulation in a variety of pathological conditions, especially cardiovascular diseases. ACE inhibitors (ACEi) and AT1R blockers (ARB) upregulate the expression of ACE2 (37–40). Potential direct activators of ACE2 are *diminazene aceturate*, r*esorcinolnaphthalein*, and *xanthenone* (41). Since SARS-CoV-2 spreads *via* the bloodstream to infect other organs, recombinant ACE2 (rhACE2) has been proposed as a therapeutic approach in pneumonia and Covid-19 (42–44). The soluble rhACE2 may be a promising approach to quench the virus when it is in the bloodstream (43, 44). However, it must be tested with caution, as soluble ACE2 is not always associated with beneficial effects (45). For instance, soluble ACE2 has a high level in men suffering from heart failure (45, 46). However, as said above, this is a condition associated with Covid-19 worsening; and this therefore suggests that soluble ACE2 may not be sufficient to protect patients. Membrane-bound ACE2 has greater anti-inflammatory effects (47).

A natural way to upregulate membrane bound ACE2 and to lower the ACE/ACE2 ratio is to exercise. It has been reported several times that physical training, and especially aerobic training may decrease ACE/Ang II, and synergistic upregulates ACE2/Ang (1-7) axis (48, 49). Although someone has put forward the hypothesis that excessive exercise is a way to increase Trojan horses (ACE2) for SARS-CoV-2 invasion, the evidence for the beneficial effects attributable to regular exercise are overwhelming.

## Discussion and Conclusions

All in all, the majority of data are in favor of the idea that a high ACE/ACE2 ratio may be detrimental for Covid-19 infection. ACE/ACE2 ratio is increased in many pathologies (especially dis-metabolisms and cardiovascular diseases) and conditions (obesity and aging) that exacerbate Covid-19 symptomatology and worsen outcomes. Moreover, ACE2 is upregulated and the ACE/ACE2 ratio is lower in many subjects at low risk for cardiovascular diseases, such as females, exercise-trained individuals, and patients well-treated with ACE inhibitors. Since most of the deceased Covid-19 patients had hypertension, further consideration is needed for ACEi and ARBs. The use of these drugs has been questioned, but the majority of authors are in favor of the use of these drugs (37–42). We agree that if used correctly they reduce the ACE / ACE2 ratio and should also be recommended to Covid-19 patients.

Are these subjects with a higher ACE2 and lower ACE/ACE2 ratio also protected against Covid-19 exacerbation? ACE2 expression could influence the course of Covid-19 in different ways: increased expression might promote viral entry, whereas ACE2 increased expression may be beneficial due to ACE2 anti-inflammatory and other beneficial effects (Figure 1) that could prevent pulmonary edema, ARDS, hypoxia, and redox stress development. It is likely that viral load is not strictly related to disease severity, and so it is likely that ACE2 overexpression is not responsible for Covid-19 worsening but that there is, rather, some other mechanism within the complex RAS or outside of RAS (such as a different macrophages population or a different immune response) that may play a role. Covid-19 is associated with an exaggerated and dysregulated systemic inflammatory response involving several inflammatory cells and leading to overproduction of several cytokines. We recently discussed in a Review article (25) the cells and the cytokines likely involved in the exacerbation of Covid-19. We pointed out how cytokine storms on cardiac and vascular endothelium may facilitate the onset of coagulopathies, thereby increasing the probability for organ ischemia and for multiple pulmonary and cardiovascular complications. The virus downregulates ACE2, exacerbating the pro-inflammatory milieu of high ACE/ACE2 ratio.

Membrane-bound ACE2 has an anti-inflammatory role, and an imbalanced and high ACE/ACE2 ratio is not recommended (Figure 1): it is better to have a low ACE/ACE2 ratio. Whether increasing the ACE2/Ang (1-7) axis by pharmacological intervention or by regular exercise may limit Covid-19 worsening remains to be ascertained. Of course, these hypotheses deserve to be studied and must be confirmed with *ad hoc* researches. Nevertheless, currently there are no effective and definitively approved drugs for the treatment of Covid-19. Therefore, understanding the molecular and cellular mechanisms that favors or exacerbates the Covid-19 in patients with altered ACE/ACE2 ratio and with comorbidities in general is urgent and necessary to design some truly effective therapies. In the meantime, we await a therapy or a vaccine; we can exercise, though we recommend to do this at home or alone to limit the diffusion of this terrible pandemic.

## Author Contributions

PP and CP made contributed to the conception and design of the work. PP drafted the work and revised it critically for important intellectual content. CP helped in finding the references and revised the manuscript critically for important intellectual content. All authors approved the version to be published.

## Conflict of Interest

The authors declare that the research was conducted in the absence of any commercial or financial relationships that could be construed as a potential conflict of interest.

## References

[1] Abassi Z Assady S Khoury EE Heyman SN. Letter to the editor: Angiotensin-converting enzyme 2: an ally or a Trojan horse? Implications to SARS-CoV-2-related cardiovascular complications. Am J Physiol Herat Circ Physiol. (2020) 318:H1080–3. 10.1152/ajpheart.00215.2020 32223552 PMC7191629

[2] Wrapp D Wang N Corbett KS Goldsmith JA Hsieh CL Abiona O. Cryo-EM structure of the 2019-nCoV spike in the prefusion conformation. Science. (2020) 367:1260–3. 10.1126/science.abb2507 32075877 PMC7164637

[3] Zhang H Penninger JM Li Y Zhong N Slutsky AS. Angiotensin-converting enzyme 2 (ACE2) as a SARS-CoV-2 receptor: molecular mechanisms and potential therapeutic target. Intensive Care Med. (2020) 46:586–90. 10.1007/s00134-020-05985-9 32125455 PMC7079879

[4] Hu Y Li X Wu N Wang N Qiu C Li J Study on the correlation among sex, age and the activity of ACE, ACE2 and the ratio of ACE/ACE2. J Qiqihar Med Univ. (2018) 39:884–7. 10.3969/j.issn.1002-1256.2018.08.005

[5] Bernardi S Toffoli B Zennaro C Tikellis C Monticone S Losurdo P. High-salt diet increases glomerular ACE/ACE2 ratio leading to oxidative stress and kidney damage. Nephrol Dial Transplant. (2012) 27:1793–800. 10.1093/ndt/gfr600 22036945

[6] Lavrentyev EN Malik KU. High glucose-induced Nox1-derived superoxides downregulate PKC-betaII, which subsequently decreases ACE2 expression and ANG(1-7) formation in rat VSMCs. Am J Physiol Heart Circ Physiol. (2009) 296:H106–18. 10.1152/ajpheart.00239.2008 18978194 PMC2637775

[7] Yuan YM Luo L Guo Z Yang M Ye RS Luo C. Activation of renin-angiotensin-aldosterone system (RAAS) in the lung of smoking-induced pulmonary arterial hypertension (PAH) rats. J Renin Angiotensin Aldosterone Syst. (2015) 16:249–53. 10.1177/1470320315576256 25795458 PMC7234796

[8] Mizuiri S Hemmi H Arita M Ohashi Y Tanaka Y Miyagi M. Expression of ACE and ACE2 in individuals with diabetic kidney disease and healthy controls. Am J Kidney Dis. (2008) 51:613–23. 10.1053/j.ajkd.2007.11.022 18371537

[9] Kehoe PG Wong S Al Mulhim N Palmer LE Miners JS. Angiotensin-converting enzyme 2 is reduced in Alzheimer's disease in association with increasing amyloid-β and tau pathology. Alzheimers Res Ther. (2016)8:50. 10.1186/s13195-016-0217-7 27884212 PMC5123239

[10] Koni I Miyamori I. Synergistic expression of angiotensin-converting enzyme (ACE) and ACE2 in human renal tissue and confounding effects of hypertension on the ACE to ACE2 ratio. Endocrinology. (2007) 148:2453–7. 10.1210/en.2006-1287 17303661

[11] Ma C Xin H Jiang XY Wang YX Zhang YS. Relationship between renal injury and the antagonistic roles of angiotensin-converting enzyme (ACE) and ACE2. Genet Mol Res. (2014) 13:2333–42. 10.4238/2014.April.3.5 24781988

[12] Mao C Liu R Bo L Chen N Li S Xia S. High-salt diets during pregnancy affected fetal and offspring renal renin-angiotensin system. J Endocrinol. (2013) 218:61–73. 10.1530/JOE-13-0139 23620529 PMC4406098

[13] Wakahara S Konoshita T Mizuno S Motomura M Aoyama C Makino Y. Enalapril protects against myocardial ischemia/reperfusion injury in a swine model of cardiac arrest and resuscitation. Int J Mol Med. (2016) 38:1463–73. 10.3892/ijmm.2016.2737 27633002 PMC5065301

[14] Wang J Li N Gao F Song R Zhu S Geng Z. Balance between angiotensin converting enzyme and angiotensin converting enzyme 2 in patients with chronic heart failure. J Renin Angiotensin Aldosterone Syst. (2015) 16:553–8. 10.1177/1470320315576257 25869724

[15] Santos SH Andrade JM Fernandes LR Sinisterra RD Sousa FB Feltenberger JD. Oral Angiotensin-(1-7) prevented obesity and hepatic inflammation by inhibition of resistin/TLR4/MAPK/NF-κB in rats fed with high-fat diet. Peptides. (2013) 46:47–52. 10.1016/j.peptides.2013.05.010 23714175

[16] Colucci JA Yuri Arita D Sousa Cunha T Seno Di Marco G Vio CP Pacheco-Silva A. Renin-angiotensin system may trigger kidney damage in NOD mice. J Renin Angiotensin Aldosterone Syst. (2011) 12:15–22. 10.1177/1470320310375456 20627940

[17] Duan K Liu B Li C Zhang H Yu T Qu J. Effectiveness of convalescent plasma therapy in severe COVID-19 patients. Proc Natl Acad Sci USA. (2020) 117:9490–6. 10.1073/pnas.2004168117 32253318 PMC7196837

[18] Hilliard LM Sampson AK Brown RD Denton KM. The “his and hers” of the renin-angiotensin system. Curr Hypertens Rep. (2013) 15:71–9. 10.1007/s11906-012-0319-y 23180053

[19] Farag NS Breitinger U Breitinger HG El Azizi MA. Viroporins and inflammasomes: a key to understand virus-induced inflammation. Int J Biochem Cell Biol. (2020) 122:105738. 10.1016/j.biocel.2020.105738 32156572 PMC7102644

[20] Rincón J Correia D Arcaya JL Finol E Fernández A Pérez M. Role of angiotensin II type 1 receptor on renal NAD(P)H oxidase, oxidative stress and inflammation in nitric oxide inhibition induced-hypertension. Life Sci. (2015) 124:81–90. 10.1016/j.lfs.2015.01.005 25623850 PMC6037991

[21] Nagata N Iwata N Hasegawa H Fukushi S Harashima A Sato Y Mouse-passaged severe acute respiratory syndrome-associated coronavirus leads to lethal pulmonary edema and diffuse alveolar damage in adult but not young mice. Am J Pathol. (2008) 172:1625–37. 10.2353/ajpath.2008.071060 18467696 PMC2408422

[22] Jia H. Pulmonary angiotensin-converting enzyme 2 (ACE2) and inflammatory lung disease. Shock. (2016)46:239–48. 10.1097/SHK.0000000000000633 27082314

[23] Dosch SF Mahajan SD Collins AR. SARS coronavirus spike protein-induced innate immune response occurs via activation of the NF-kappaB pathway in human monocyte macrophages *in vitro*. Virus Res. (2009) 142:19–27. 10.1016/j.virusres.2009.01.005 19185596 PMC2699111

[24] Tan SY Krasnow MA. Developmental origin of lung macrophage diversity. Development. (2016) 143:1318–27. 10.1242/dev.129122 26952982 PMC4852511

[25] Moccia F Gerbino A Lionetti V Miragoli M Munaron LM Pagliaro P. COVID-19-associated cardiovascular morbidity in older adults: a position paper from the Italian Society of Cardiovascular Researches. Geroscience. (2020) 20:1–29. 10.1007/s11357-020-00198-w 32430627 PMC7237344

[26] Ma Y Mouton AJ Lindsey ML. Cardiac macrophage biology in the steady-state heart, the aging heart, and following myocardial infarction. Transl Res. (2018) 191:15–28. 10.1016/j.trsl.2017.10.001 29106912 PMC5846093

[27] Oudit GY Kassiri Z Jiang C Liu PP Poutanen SM Penninger JM. SARS-coronavirus modulation of myocardial ACE2 expression and inflammation in patients with SARS. Eur J Clin Invest. (2009) 39:618–25. 10.1111/j.1365-2362.2009.02153.x 19453650 PMC7163766

[28] Ramchandran R Pilipenko E Bach L Raghavan A Reddy SP Raj JU. Hypoxic regulation of pulmonary vascular smooth muscle cyclic guanosine monophosphate-dependent kinase by the ubiquitin conjugating system. Am J Respir Cell Mol Biol. (2012) 46:323–30. 10.1165/rcmb.2011-0165OC 21997485 PMC3326432

[29] Gassmann M Muckenthaler MU. Adaptation of iron requirement to hypoxic conditions at high altitude. J Appl Physiol. (1985) 119:1432–40. 10.1152/japplphysiol.00248.2015 26183475

[30] Ando T Mikawa K Nishina K Misumi T Obara H. Hypocapnic alkalosis enhances oxidant-induced apoptosis of human alveolar epithelial type II cells. J Int Med Res. (2007) 35:118–26. 10.1177/147323000703500113 17408063

[31] Kreü S Jazrawi A Miller J Baigi A Chew M. Alkalosis in critically Ill patients with severe sepsis and septic shock. PLoS ONE. (2017) 12:e0168563. 10.1371/journal.pone.0168563 28045915 PMC5207677

[32] Mæhle K Haug B Flaatten H Nielsen E. Metabolic alkalosis is the most common acid–base disorder in ICU patients. Crit Care. (2014) 18:420. 10.1186/cc13802 25001067 PMC4056091

[33] Ottolenghi S Rubino FM Sabbatini G Coppola S Veronese A Chiumello D. Oxidative stress markers to investigate the effects of hyperoxia in anesthesia. Int J Mol Sci. (2019) 20:E5492. 10.3390/ijms20215492 31690051 PMC6862279

[34] Ottolenghi S Sabbatini G Brizzolari A Samaja M Chiumello D. Hyperoxia and oxidative stress in anesthesia and critical care medicine. Minerva Anestesiol. (2020) 86:64–75. 10.23736/S0375-9393.19.13906-5 31680497

[35] Damiani E Donati A Girardis M Oxygen in the critically ill: friend or foe? Curr Opin Anaesthesiol. (2018) 31:129–35. 10.1097/ACO.0000000000000559 29334496

[36] Chen J Jiang Q Xia X Liu K You Z Tao W Individual variation of the SARS-CoV2 receptor ACE2 gene expression and regulation. Preprints. (2020). Available online at: https://www.preprints.org/manuscript/202003.0191/v1 10.1111/acel.13168 PMC7323071 32558150

[37] Saavedra JM. Angiotensin receptor blockers and COVID-19. Pharmacol Res. (2020) 156:104832. 10.1016/j.phrs.2020.104832 32304747 PMC7158830

[38] Arendse LB Danser AHJ Poglitsch M Touyz RM Burnett JC Jr Llorens-Cortes C. Novel therapeutic approaches targeting the renin-angiotensin system and associated peptides in hypertension and heart failure. Pharmacol Rev. (2019) 71:539–70. 10.1124/pr.118.017129 31537750 PMC6782023

[39] Deshotels MR Xia H Sriramula S Lazartigues E Filipeanu CM. Angiotensin II mediates angiotensin converting enzyme type 2 internalization and degradation through an angiotensin II type I receptor–dependent mechanism. Hypertension. (2014) 64:1368–75. 10.1161/HYPERTENSIONAHA.114.03743 25225202 PMC4231883

[40] Zores F Rebeaud ME COVID and the renin-angiotensin system: are hypertension or its treatments deleterious? Front Cardiovasc Med. (2020) 23:71 10.3389/fcvm.2020.00071 PMC7191060 32391384

[41] Li Y Zhou W Yang L You R. Physiological and pathological regulation of ACE2, the SARS-CoV-2 receptor. Pharmacol Res. (2020) 14:104833. 10.1016/j.phrs.2020.104833 32302706 PMC7194807

[42] Khan A Benthin C Zeno B Albertson TE Boyd J Christie JD. A pilot clinical trial of recombinant human angiotensin-converting enzyme 2 in acute respiratory distress syndrome. Crit Care. (2017) 21:234. 10.1186/s13054-017-1823-x 28877748 PMC5588692

[43] Monteil V Kwon H Prado P Hagelkrüys A Wimmer RA Stahl M. Inhibition of SARS-CoV-2 infections in engineered human tissues using clinical-grade soluble human ACE2. Cell. (2020) 181:905–913.e7. 10.1016/j.cell.2020.04.004 32333836 PMC7181998

[44] Ciaglia E Vecchione C Puca AA. COVID-19 infection and circulating ACE2 levels: protective role in women and children. Front Pediatr. (2020) 8:206. 10.3389/fped.2020.00206 32391299 PMC7192005

[45] Patel VB Zhong JC Grant MB Oudit GY. Role of the ACE2/angiotensin 1-7 axis of the renin-angiotensin system in heart failure. Circ Res. (2016) 118:1313–26. 10.1161/CIRCRESAHA.116.307708 27081112 PMC4939482

[46] Sama IE Ravera A Santema BT van Goor H Ter Maaten JM Cleland JGF. Circulating plasma concentrations of angiotensin-converting enzyme 2 in men and women with heart failure and effects of renin-angiotensin-aldosterone inhibitors. Eur Heart J. (2020) 41:1810–7. 10.1093/eurheartj/ehaa373 32388565 PMC7239195

[47] Verdecchia P Cavallini C Spanevello A Angeli F. The pivotal link between ACE2 deficiency and SARS-CoV-2 infection. Eur J Intern Med. (2020) 76:14–20. 10.1016/j.ejim.2020.04.037 32336612 PMC7167588

[48] Keidar S Kaplan M Gamliel-Lazarovich A. ACE2 of the heart: from angiotensin I to angiotensin (1-7). Cardiovasc Res. (2007)73:463–9. 10.1016/j.cardiores.2006.09.006 17049503

[49] Fernandes T Hashimoto NY Magalhães FC Fernandes FB Casarini DE Carmona AK. Aerobic exercise training-induced left ventricular hypertrophy involves regulatory MicroRNAs, decreased angiotensin-converting enzyme-angiotensin ii, and synergistic regulation of angiotensin-converting enzyme 2-angiotensin (1-7). Hypertension. (2011) 58:182–9. 10.1161/HYPERTENSIONAHA.110.168252 21709209 PMC3184458

